# Allelic Differentiation at the *E1/Ghd7* Locus Has Allowed Expansion of Rice Cultivation Area

**DOI:** 10.3390/plants8120550

**Published:** 2019-11-28

**Authors:** Hiroki Saito, Yutaka Okumoto, Takuji Tsukiyama, Chong Xu, Masayoshi Teraishi, Takatoshi Tanisaka

**Affiliations:** 1 Graduate School of Agriculture, Kyoto University, Kyoto, Kyoto 606-8502, Japan; okumoto.yutaka.4w@kyoto-u.ac.jp (Y.O.); tsukiyama@nara.kindai.ac.jp (T.T.); xuchong@kiui.ac.jp (C.X.); temple@kais.kyoto-u.ac.jp (M.T.); t_tanisa@kiui.ac.jp (T.T.); 2 Tropical Agriculture Research Front, Japan International Research Center of Agricultural Science, Ishigaki, Okinawa 907-0002, Japan; 3 Faculty of Agriculture, Kindai University, Nara, Nara 631-8505, Japan; 4 School of Agriculture, Kibi International University, Minami-Awaji 656-0484, Japan

**Keywords:** rice, flowering time, photoperiod sensitivity, allelic variation, fine-tuning

## Abstract

The photoperiod-insensitivity allele *e1* is known to be essential for the extremely low photoperiod sensitivity of rice, and thereby enabled rice cultivation in high latitudes (42–53° north (N)). The *E1* locus regulating photoperiod-sensitivity was identified on chromosome 7 using a cross between T65 and its near-isogenic line T65w. Sequence analyses confirmed that the *E1* and the *Ghd7* are the same locus, and haplotype analysis showed that the *e1/ghd7-0a* is a pioneer allele that enabled rice production in Hokkaido (42–45° N). Further, we detected two novel alleles, *e1-ret/ghd7-0ret* and *E1-r/Ghd7-r*, each harboring mutations in the promoter region. These mutant alleles alter the respective expression profiles, leading to marked alteration of flowering time. Moreover, *e1-ret/ghd7-0ret*, as well as *e1/ghd7-0a*, was found to have contributed to the establishment of Hokkaido varieties through the marked reduction effect on photoperiod sensitivity, whereas *E1-r/Ghd7-r* showed a higher expression than the *E1/Ghd7* due to the nucleotide substitutions in the *cis* elements. The haplotype analysis showed that two photoperiod-insensitivity alleles *e1/ghd7-0a* and *e1-ret/ghd7-0ret*, originated independently from two sources. These results indicate that naturally occurring allelic variation at the *E1/Ghd7* locus allowed expansion of the rice cultivation area through diversification and fine-tuning of flowering time.

## 1. Introduction

Rice is a major cereal extensively cultivated in a wide range of latitudes from 55° N to 35° S. Because rice is formerly a facilitative short-day (SD) plant well adapted to warm climate, photoperiodic control of flowering time is a key factor in the regional and seasonal adaptability of rice varieties [1]. In high latitudes (>ca. 40° N), rice cultivation had been impracticable due to the short summer and long-day (LD) more than 15 h during the summer, until early flowering varieties with extremely weak photoperiod sensitivity were raised [2,3,4]. It was during 1900 to 1930 that such varieties were first released and planted in the northernmost rice cultivation area, Hokkaido, in Japan (42–45° N) [5]. The varieties raised for Hokkaido also enabled rice cultivation even in Hei Long Jiang province (43–53° N) of China [6].

Also in low latitudes (ca. 20° S–20° N), a recent rice breeding program aims to produce varieties with weak photoperiod sensitivity (PS), though a long basic vegetative growth period is necessary at the same time, because such a combination of the two traits for heading will permit almost constant and adequate vegetative growth periods under SD (less than 13.5 h) [7,8,9]. In addition, in middle latitudes (30–40° N), there is a close relation between the photoperiod sensitivity of varieties and the latitude of their cultivation area [3,4]. Thus, understanding of the genetic factors responsible for photoperiod sensitivity, as well as basic vegetative growth, will be essential for not only guaranteeing stable rice production but also allowing further expansion of rice cultivation area. 

Genetic studies on rice flowering (heading) time started in 1915 [10]. Since then, many flowering time loci were reported: among them, *E1* [11,12,13,14], *Photosensitivity 1* (*Se1*) [15], and *Earliness 1* (*Ef1*) [16], have been intensively studied about their genetic characteristics, such as allelic variation, response to photoperiod, geographical distribution, and interaction with other loci. The geographical studies showed that these three loci play especially important roles in regional adaptabilities of Japanese and Taiwanese *japonica* rice varieties and *japonica*/*indica* cross varieties in Korea [4,5,6,15,17,18,19,20,21,22,23].

The Committee on Gene Symbolization, Nomenclature and Linkage Groups of the Rice Genetics Cooperative made a rule that the gene symbols which have been commonly used by many workers in the past should be retained [24], and recommended to categorize flowering time genes into three types, earliness and lateness (gene symbol: *E*), photoperiod sensitivity (gene symbol: *Se*), and basic vegetative growth (gene symbol: *Ef*). With the advance of quantitative trait locus (QTL) analysis, however, it has become difficult to categorize newly found QTLs into three types because they are detected only from flowering time data. Since then, the gene symbols, *E*, *Se*, and *Ef*, did not come to be retained. Recent molecular genetic analyses identified three key flowering time loci, *Heading date 1* (*Hd1*) [25], *Early heading date 1* (*Ehd1*) [26], and *Grain number, plant height, and heading date 7* (*Ghd7*) [27], all of which were named regardless of the rule, and subsequent studies on these three loci provided new numerous molecular-based knowledges of rice flowering. Similarly, about the *E1*, *Se1* and *Ef1* loci, the information useful for rice breeding has been accumulated with enormous numbers until now. Therefore, it is significant to clarify the relationships of the three loci, *E1*, *Se1* and *Ef1*, to the loci named regardless of the rule. To date, the *Se1* and *Ef1* loci proved to be identical with the *Hd1* [21,28] and the *Ehd1* loci [7], respectively. 

The *E1* was first identified as a late flowering time locus: the functional allele *E1* is completely dominant over the nonfunctional allele *e1* [11,12]. This locus was also involved in plant height. Later, this locus proved to control PS [13], and its functional allele *E1* was shown essentially important in rice varieties for temperate areas in Japan (30–40° N) [17,18] because of firmly inhibiting the panicle primordial differentiation under LD until it becomes SD conditions, and thereby ensuring normal vegetative growth and stable yields. In contrast, a photoperiod-insensitivity allele *e1* was found to be essential for the varieties commercially cultivated in Hokkaido (42–45° N), because of its marked reducing effect on photoperiod sensitivity: use of *e1* enabled rice cultivation in high latitudes where LD conditions continue during the summer [5,21]. The *E1* locus was found to be located on chromosome 7, linked to the *rfs* (rolled fine strip) and *slg* (slender glume) loci with recombination values of 16.3% and 9.1%, respectively [29]. This locus has been well investigated for its effects on photoperiod sensitivity and regional adaptabilities of rice plants [5,11,12,13,14,15,16,17,18,19], but little is known about the relationship with the loci which were identified by molecular genetic analysis and named regardless of the rule. Recently, the *Ghd7* locus was precisely mapped on chromosome 7, and this locus exert major effects on not only heading date but also number of grains per panicle and plant height [27]. In addition, subsequent molecular analyses of the *Ghd7* locus demonstrated that a loss-of-function allele of *Ghd7* is essential for the extremely early flowering of Hokkaido varieties [30,31,32]. These reports make a conjecture that the *E1* and *Ghd7* are the same locus.

In the present study, we first analyzed the effects of three alleles at the *E1* locus on photoperiod sensitivity using the Taiwanese *japonica* rice variety “Taichung 65 (T65)” harboring *E1* [33], its isogenic line T65m harboring *e1* [16,33,34,35], and T65w that harbors a chromosome segment of *O. rufipogon* Griff. including the *E1* locus in the genetic background of T65 [36]. Subsequently, we attempted to determine the precise chromosomal location of the *E1* locus using the progenies from T65 × T65w, and then conducted sequence analysis to learn the sequences of the three alleles, also to investigate the relationship between the two loci, *E1* and *Ghd7*. We finally applied a haplotype analysis of the chromosomal region surrounding the *E1/Ghd7* locus to 44 Hokkaido and 50 Japanese-core-collection varieties in order to prove correctness of the findings by Okumoto et al. (1996) [5] and Ichitani et al. (1998) [21] that *e1* is the key allele for establishing the varieties for the northernmost rice cultivation area, and its history and origin. 

## 2. Results

### 2.1. Photoperiod Sensitivities of T65, T65w, and T65m

**Figure 1:**
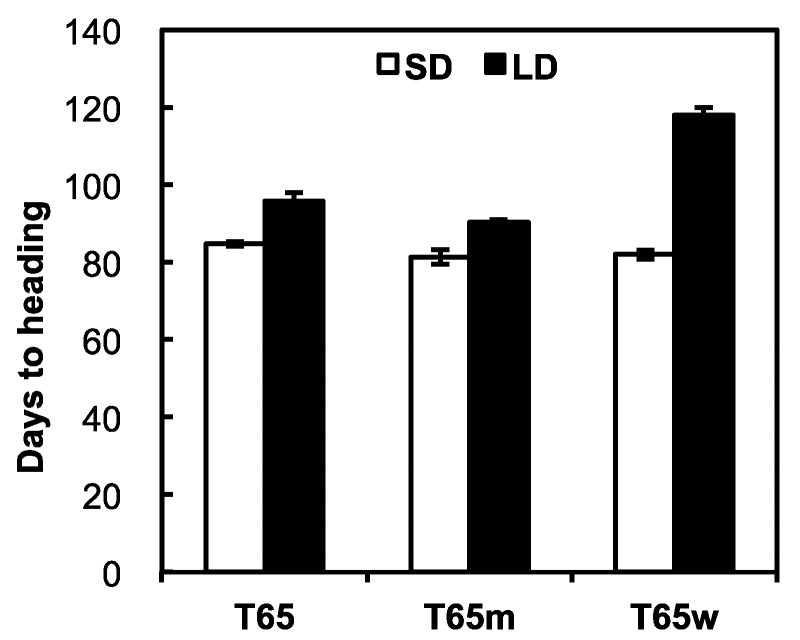
Days to heading of T65, T65m, and T65w under short-day (SD, white bar) and long-day (LD, black bar) conditions.

Days to heading (DH) of T65, T65m, and T65w under a SD were 84.7, 81.4, and 82.0 respectively, while those under a LD were 95.6, 90.4, and 118.0, respectively (Figure 1). Thus, the photoperiod sensitivities of T65, T65m, and T65w were estimated at 11.1, 9.0, and 36.0, respectively. Since T65m is an isogenic line of T65 for the *E1* locus, the weaker PS of T65m was attributable to the photoperiod-insensitivity allele e1 at the *E1* locus. T65w showed far stronger photoperiod sensitivity than T65 and T65m. The genotypic difference between T65w and T65 is only in the chromosome region including the *E1* locus, where only T65w harbors a chromosome segment induced from *O. rufipogon* Griff. Since any other photoperiod sensitivity genes have not yet been reported in this region, we conclude that the chromosome segment introduced from *O. rufipogon* Griff. in T65w certainly harbors a strong photoperiod-sensitivity allele, probably at the *E1* locus.

### 2.2. Chromosomal Location of the E1 Locus

**Figure 2:**
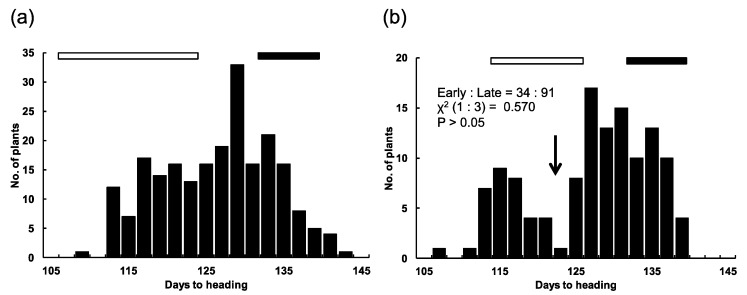
Distributions of days to heading in two F_2_ populations from crosses between (**a**) T65 × T65w and (**b**) T65w × T65m. The black bar indicates the range of days to heading of T65w. The white bar indicates the ranges of days to heading of (**a**) T65 and (**b**) T65m. The arrow indicates the breakpoint between early and late heading groups.

The F_2_ population from the cross between T65 and T65w, comprising 205 plants, showed a continuous distribution of DH within the parental ranges (Figure 2a). We conducted a progeny test using 38 F_3_ lines, which were derived from randomly selected F_2_ plants. In the test, all the F_3_ lines were clearly classified into three groups. The ratio of [T65-type]:[segregating-type]:[T65w-type] lines was 13:14:11, which fitted the 1:2:1 ratio expected for one-locus segregation (χ^2^ = 8.904, P > 0.05) (Table S1). In contrast, the F_2_ population from the cross between T65w and T65m showed a bimodal distribution of DH within the parental ranges, with a clear breakpoint dividing the population into early (T65m-type) and late (T65w-type) groups (Figure 2b). The ratio of early type (34 plants): late type (91 plants) fitted the 1:3 ratio expected for one-locus segregation (χ^2^ = 0.570, P > 0.05). In the progeny test, all the 40 F_3_ lines were clearly classified into three groups. The ratio of [T65m type]:[segregating type]:[T65w type] lines fitted the 1:2:1 ratio expected for one-locus segregation (χ^2^ = 0.150, P > 0.05) (Table S2). T65m is an isogenic line of T65 harboring a recessive allele *e1* at the *E1* locus. We accordingly inferred that T65w harbors a novel allele at the *E1* locus, whose heading-date delaying effect was stronger than *E1* in T65. We designated this allele *E1-r* (a novel photoperiod-sensitivity allele at the *E1* locus). 

**Figure 3:**
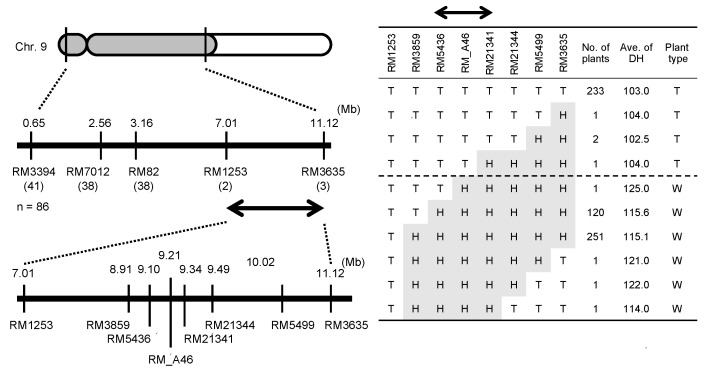
Map-based cloning and graphical genotypes of the candidate region of the E1 locus. “T” and “H” at each marker indicate T65 homozygous and heterozygous, respectively. “T” and “W” at plant type indicate T65-type (early heading) and T65w-type (late heading), respectively.

Using 546 F_3_ plants from the cross between T65 and T65w, we tried to identify the chromosomal location of the *E1* locus. The result showed that the *E1* locus was present in the region with a physical distance of 4.11 Mb between two simple sequence repeat (SSR) markers, RM1253 and RM3635, on chromosome 7 (Figure 3). Subsequently, we attempted to narrow down the candidate region of the *E1* locus, using 1263 F_4_ progenies derived from several F_3_ recombinants between RM1253 and RM3635; consequently, the chromosomal location of the *E1* locus was narrowed down to the region with a physical length of approximately 228.1-kb between RM5436 and RM21341 (Figure 3). In this region, 11 genes are reported in Rice Annotation Program Database [37]. Among them, we proposed that Os07g0261200, which was reported as *Ghd7*, a repressor of flowering time under LD conditions [27], was likely to be a candidate of *E1*.

**Figure 4:**
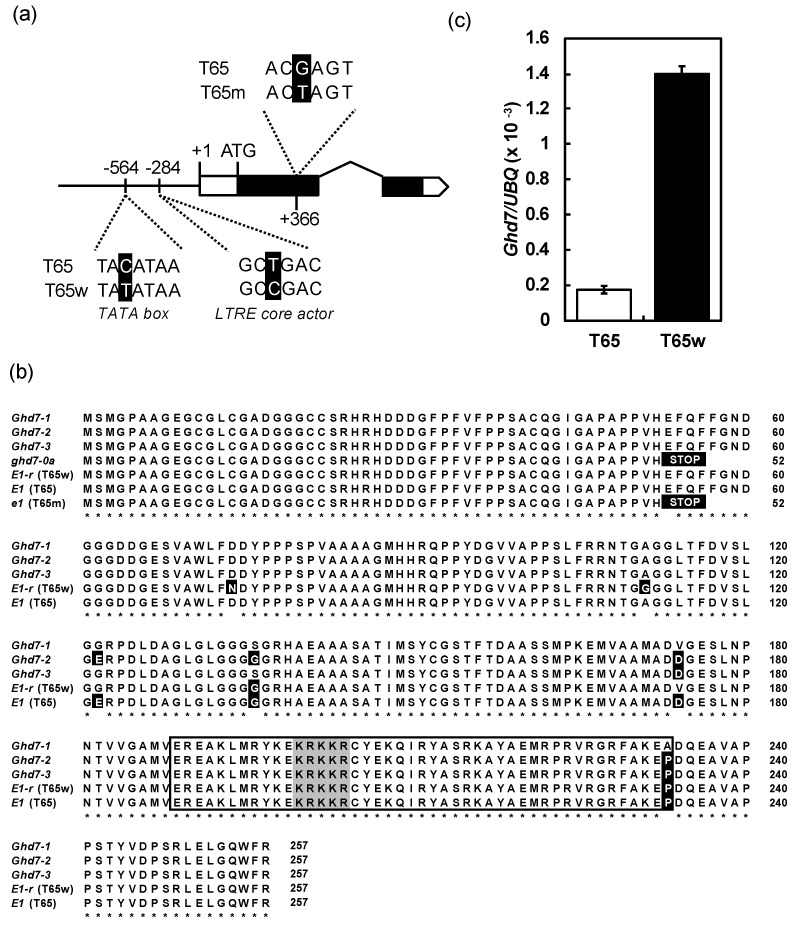
(**a**) Schematic diagrams of the alleles at the *E1/Ghd7* focused on nucleotide substitutions among T65 T65m, and T65w. (**b**) Alignments of amino acid sequences of the alleles at the *E1/Ghd7* locus. The white and black characters with black and gray cells indicate amino acid substitutions and CONSTANS, CO-like, and TOC1 (CCT)-motif, respectively. The box indicates the CCT-motif region. *Ghd7-1*, *Ghd7-2* and *Ghd7-3* were functional alleles [29]. (**c**) Comparison of the expression level of the allele at the *E1/Ghd7* locus between T65 and T65w.

Sequence analyses showed that the sequences of the alleles at the *Ghd7* locus in T65 and T65m were completely consistent with a functional allele *Ghd7-2* [38] and a nonfunctional allele *ghd7-0a* [27], respectively (Figure 4a,b). Since the genotypic difference in flowering time between T65 and T65m is only at the *E1* locus, this suggests that *E1* and *Ghd7* are the same locus (hereafter we tentatively designate *E1* (=*Ghd7*) as *E1/Ghd7*), and that T65m flowered earlier than T65 because the former harbors a loss-of-function allele *e1/ghd7-0a*. In contrast, the allele of T65w at the *E1* locus harbored four nonsynonymous substitutions and two nucleotide substitutions in the promoter region (Figure 4a,b). Among the substitutions, two in the promoter region were in the transcriptional signal motifs (cis elements): low temperature response element (LTRE) core actor (located at −284) and the TATA box (located at −564). Thus, the two nucleotide substitutions were considered to modify the expression of *E1/Ghd7*. Subsequent expression analysis of *E1/Ghd7* showed that the expression of T65w was higher than that of T65 (Figure 4c). This suggests that the late flowering of T65w is caused by high expression of *E1/Ghd7* due to the nucleotide substitutions in the cis elements.

### 2.3. A Novel Nonfunctional Allele at the E1/Ghd7 Locus 

**Figure 5:**
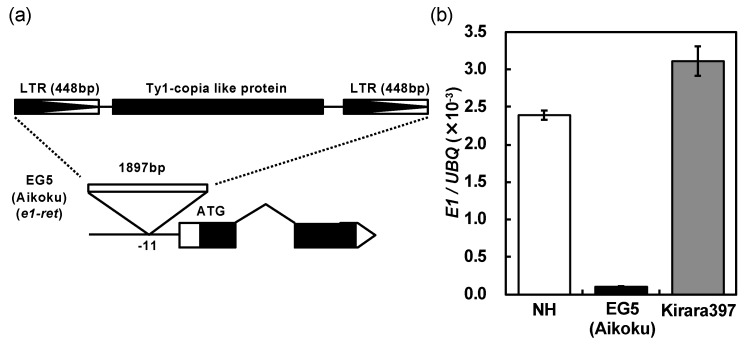
(**a**) Schematic diagram of the allele at the *E1/Ghd7* locus in “EG5 (Aikoku)”. (**b**) Comparison of the expression level of the allele at the *E1/Ghd7* locus among three Japanes varieties “Nipponbare” (NH), “EG5 (Aikoku)” and “Kirara397”. “EG5 (Aikoku)” and “Kirara397” are a tester line for the *E1*, *E2* and *E3* locus and an elite Hokkaido variety, respectively.

Okumoto et al. (1996) [5] showed that nine Hokkaido varieties tested all harbored a nonfunctional (photoperiod-insensitivity) allele *e1* at the *E1* locus thorough a conventional genetic analysis, and assumed that this allele has played an essential role in the establishment of rice varieties for the Hokkaido district. To confirm this assertion, we analyzed the presence of the nucleotide substitution from GAG (Glu) to TAG (stop codon) in exon 1 at the *E1/Ghd7* locus (Figure 4a) of 44 Hokkaido varieties using a cleaved amplified polymorphic sequence (CAPS) marker. The result showed that 37 varieties harbored the *e1/ghd7-0a* allele, and 7 varieties did not (Table S3). This single nucleotide substitution was not observed in EG5 (Aikoku), which is one of the tester lines for the *E1*, *E2* and *E3* loci involved in the flowering time, and which harbors *e1* allele at the *E1* locus [11,12,13]. Sequence analysis for the EG5 revealed that a Ty1-copia like retrotransposon (TE) was inserted in the promoter region of the *E1/Ghd7* allele (Figure 5a). The seven varieties, which did not harbor the *e1/ghd7-0a* allele, also harbored the same TE insertion. We named this novel nonfunctional allele *e1-ret/ghd7-0ret*. The expression of the *e1-ret/ghd7-0ret* allele was far lower than the *E1/Ghd7-2* allele in the Japanese variety “Nipponbare” with the reference genome (Figure 5b), indicating that *e1-ret/ghd7-0ret* confers extremely weak photoperiod sensitivity by losing the normal function of the promoter.

### 2.4. Haplotype Patterns of the Chromosomal Region Surrounding E1/Ghd7 Locus

**Figure 6:**
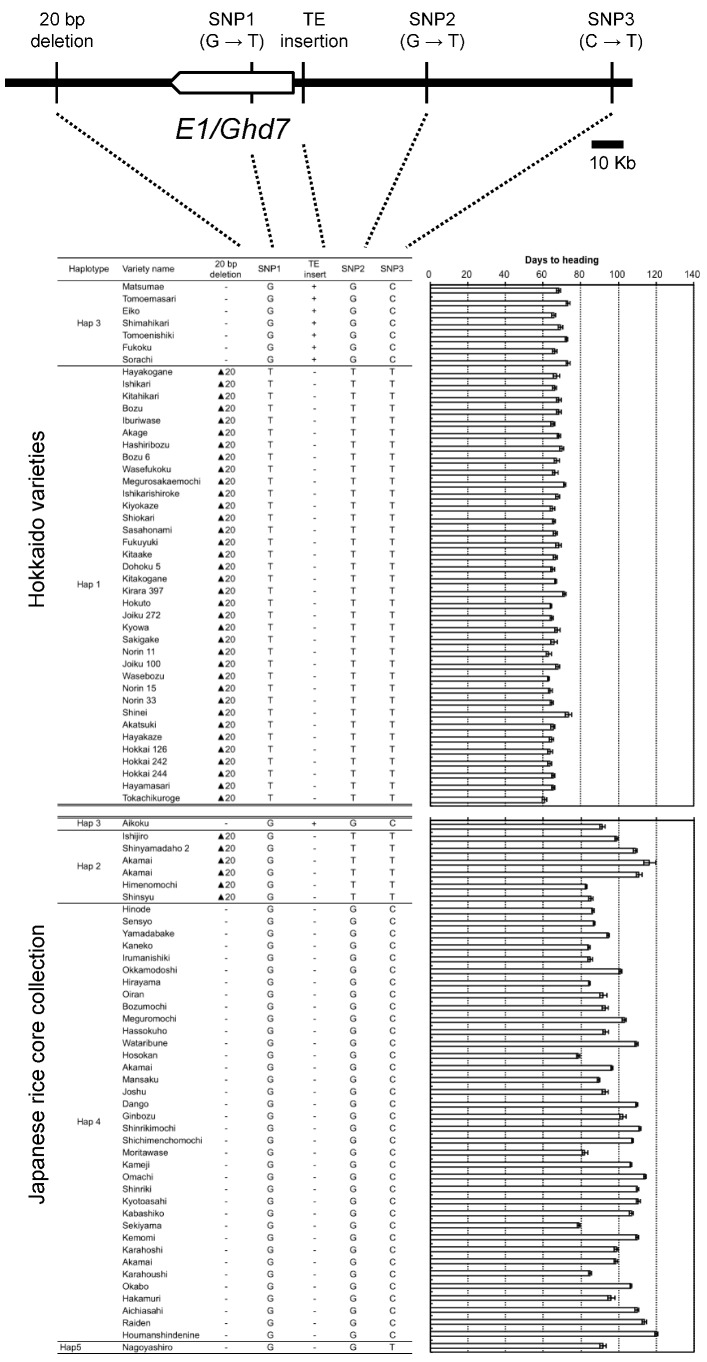
Haplotypes around the *E1/Ghd7* locus and days to heading of Hokkaido and Japanese core collection varieties.

**Figure 7:**
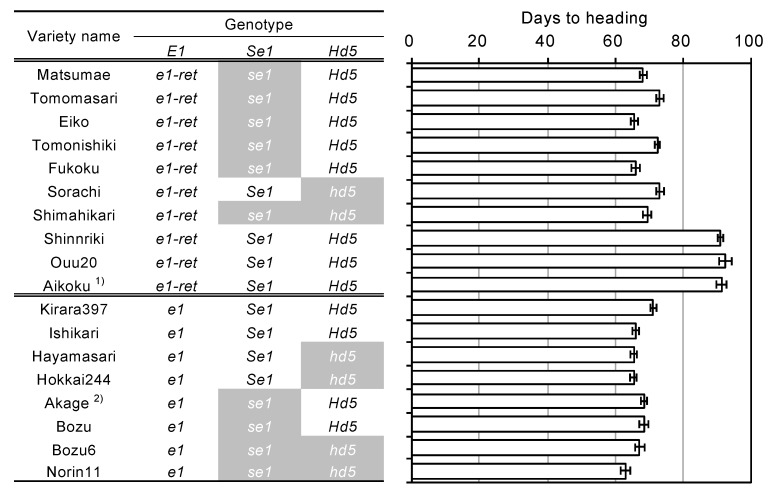
Gene combinations for the *E1*, *Se1*, and *Hd5* loci and days to heading. ^1)^ This “Aikoku” variety belongs to the Japanese core collections. ^2)^ This “Akage” variety belongs to the Hokkaido varieties.

We surveyed DNA polymorphisms between EG5 (Aikoku) (*e1-ret/ghd7-0ret*) and Kirara397 (*e1/ghd7-0a*) around *E1/Ghd7* locus. Subsequently, we found three polymorphisms (two SNPs and a 20-bp deletion) other than the nucleotide substitution from GAG (Glu) to TAG (stop codon) and the TE insertion. To know the origins of two nonfunctional alleles *e1/ghd7-0a* and *e1-ret/ghd7-0ret*, we investigated the haplotypes of Hokkaido and Japanese-core collection varieties using five markers surrounding the *E1/Ghd7* locus (three single nucleotide polymorphisms (SNPs), a 20-bp deletion, and a TE insertion). The Japanese-core-collection varieties were classified into at least four haplotypes, Hap2, Hap3, Hap4, and Hap5 (Figure 6 and Table S4). In contrast, Hokkaido varieties were classified into two distinct haplotypes, Hap1 and Hap3. This suggests that Hap1 was derived from Hap2 via nucleotide substitution from GAG (Glu) to TAG (stop codon) in exon 1, whereas Hap3 was derived from Hap4 via the TE insertion in the promoter region. These results indicate that two independent mutational events contributed to the occurrence of the two nonfunctional alleles *e1/ghd7-0a* and *e1-ret/ghd7-0ret*. Interestingly, although Hap1 was found only in Hokkaido varieties, Hap3 was found not only in Hokkaido varieties but also in some Japanese varieties, particularly in the Aikoku-related varieties (Figure 6 and Table S4). Further, the varieties of the Hap3 group, except for Hokkaido varieties, flowered about 20 days later than the Hokkaido varieties, implying that such varieties do not adapt to Hokkaido where autumn comes early (Figure 6 and Table S4). These findings indicate that other genetic factor (s) were involved in the early flowering of Hokkaido varieties belonging to the Hap3 group. We accordingly investigated allelic variations in the *Se1/Hd1* and another major photoperiod-sensitivity gene, *Hd5*, which is known to be involved in the PS in the Hokkaido varieties [30,31,32]. The results showed that varieties with *e1/ghd7-0a* flowered early regardless of harboring a functional allele(s) (photoperiod-sensitivity allele) at the *Se1/Hd1* and/or *Hd5* locus, whereas varieties with *e1-ret/ghd7-0ret* flowered early only when harboring a nonfunctional allele at either of the *Se1/Hd1* or *Hd5* locus (Figure 7). These results indicate that coexistence of *e1-ret/ghd7-0ret* with a photoperiod-insensitivity allele either at the *Se1/Hd1* or at the *Hd5* locus is necessary to promote flowering under LD conditions.

## 3. Discussion

Since Hoshino (1915) [10], many genes (loci) controlling flowering time have been reported (reviewed in [39,40,41]). Among them, the *E1* is an important locus closely associated with the regional adaptability of rice varieties: its photoperiod insensitivity allele *e1* enabled rice cultivation even in Hokkaido, one of the northernmost rice cultivation area (42–45° N) [5,13,21]. A dominant photoperiod-sensitivity allele *E1* at the *E1* locus widely deployed among Japanese varieties for all regions other than Hokkaido in Japan [17,18,21]. Ichitani et al. (1998) [21] reported that the *E1* locus was identical to the *Heading date 4* (*Hd4*) locus, which was identified by Quantitative Trait Locus (QTL) analysis of flowering time using progenies from the cross of the *indica* variety Kasalath and the *japonica* variety Nipponbare [25]. Fujino and Sekiguchi (2005) [30] identified two QTLs, *qDTH-7-1* and *qDTH-7-2*, for flowering time using progenies from the cross between two Hokkaido varieties, Hoshinoyume and Nipponbare. They concluded that *qDTH-7-1* is the same locus as the *E1* (*Hd4*). Later, Xue et al. (2008) [27] isolated *the grain number, plant height, and heading date 7* (*Ghd7*) locus on chromosome 7, whose functional allele *Ghd7* encodes a *CONSTANS*, *CO-like*, and *TOC1* (CCT) domain-containing protein that delays flowering under LD conditions. They also reported that a nonfunctional allele *ghd7-0a*, harboring a premature termination in the predicted coding region, deployed among varieties commercially cultivated in Hei Long Jiang province, China (43–53° N). In the present study, we successfully determined the chromosomal location of the *E1* locus within a 228.1-kb physical region on chromosome 7 (Figure 3). According to the rice public database RAP-DB [38], 11 loci (genes) exist in this region. Among the genes, only Os07g0261200 (=*Ghd7*) showed a SNP in exon 1 of a photoperiod insensitive allele *e1* in T65m (Figure 4). This substitution was the same as that of the nonfunctional allele *ghd7-0a* [27]. From these results, we concluded that the *E1*, *Hd4*, *qDTH-7-1* and *Ghd7* are the same locus. Then we finally designate this locus as *E1/Ghd7*. It is noteworthy that we identified a novel photoperiod-insensitivity allele *e1-ret/ghd7-0ret* that harbored a TE insertion in the promoter region of *E1/Ghd7*. We conclude that this TE-insertional mutation causes the loss of function of the *E1/Ghd7* allele. 

In addition, we identified a novel strong photoperiod-sensitivity allele *E1-r/Ghd7-r*, which harbors three nonsynonymous substitutions in the coding sequence (CDS) and two SNPs in the promoter region: one is in the LTRE core actor (CCGAC), and the other is in the TATA-box (Figure 5a). The LTRE core actor was identified in the regulatory regions of all cold-induced genes in Arabidopsis [42]. The CCGAC core motif, also known as C-repeat / drought response element (CRT/DRE), is essential for transcriptional activation in response to cold, drought, and/or high-salt treatments [43]. Kim et al. (2002) [44] showed that light signaling mediated by phytochrome activates cold-induced gene expression through CRT/DRE. The expression of *E1-r/Ghd7-r* was higher than that of a PS allele of *E1/Ghd7-2* in Nipponbare (Figure 5b). The expression level of *E1/Ghd7* is regulated by red light signal and correlated well with its LD specific activity [45,46]. These suggest that the mutations in the promoter region modify the *E1-r/Ghd7-r* expression, which delays flowering under LD conditions. Elucidation of the influences of the three amino acid substitutions in the CDS of *E1-r/Ghd7-r* awaits further study.

Haplotype analysis showed that two photoperiod-insensitive alleles, *e1/ghd7-0a* and *e1-ret/ghd7-0ret*, originated independently from two sources (Figure 6). *e1/ghd7-0a* widely deployed among improved and landrace varieties in Hokkaido, including “Akage”, which was one of the pioneers of the Hokkaido rice varieties in the late 1800’s [47]. This indicates that *e1/ghd7-0a* is a pioneer allele, leading to raising extremely early heading varieties with extremely weak photoperiod sensitivity during 1900–1930 (see Introduction). In contrast, *e1-ret/ghd7-0ret* was detected in “Fukoku”, one of the past leading varieties in Hokkaido. “Fukoku” was bred from the cross between the Japanese warm region variety “Nakate-Aikoku”, and the Hokkaido variety “Bozu 6”. “Nakate-Aikoku” harbors *e1-ret/ghd7-0ret*, whereas “Bozu 6” does not. This indicates that *e1-ret/ghd7-0ret* of “Fukoku” was derived from “Nakate-Aikoku” (Figure S1). Interestingly, although most of the Aikoku-related varieties harbor *e1-ret/ghd7-0ret* (Table S4), none of them flowered as early as “Fukoku”. This indicates that some genetic factor(s) other than *e1-ret/ghd7-0ret* is also responsible for the early flowering of “Fukoku”. The varieties with *e1-ret/ghd7-0ret* flowered extremely early when harboring a nonfunctional allele either at the *Se1/Hd1* locus or at the *Hd5* locus (Figure 7). Therefore, we conclude that a nonfunctional allele either at the *Se1/Hd1* locus or at the *Hd5* locus is necessary for early flowering of the Hokkaido varieties with *e1-ret/ghd7-0ret*. Fujino et al. (2013) [32] reported that the nonfunctional allele at the *Hd5* locus was a spontaneous mutant gene that occurred in the Hokkaido local landrace “Bozu,” and that this allele contributed to the expansion of rice cultivation to the northern area of Hokkaido. In contrast, the nonfunctional allele at the *Se1/Hd1* locus widely deployed among the varieties for the Tohoku-Hokuriku region (37–40° N) [15,18]. In the early rice breeding in Hokkaido, many Aikoku-related varieties, which were chiefly cultivated in the Tohoku-Hokuriku region, Japan, were frequently used as cross-parents to increase the genetic diversity and improve grain quality [47]. This suggests that *e1-ret/ghd7-0ret* was introduced from the Aikoku-related varieties, and the combination with the nonfunctional allele at the *Hd1* or *Hd5* locus made *e1-ret/ghd7-0ret* available for rice breeding programs in Hokkaido. It is also suggested that mutations in the promoter region often make functional differentiations of alleles at the *E1/Ghd7* locus, bringing about flowering time diversification in rice. 

Recent molecular genetic studies revealed that there are large natural allelic variations in key loci controlling flowering time, such as *Ghd7*, *Hd1*, *DTH2*, and *DTH7*, which contribute to the diversity of flowering time, and to the regional adaptability by adjusting flowering time to distinct environmental conditions [27,32,48,49,50,51]. Zhang et al. (2015) [52] and Zheng et al. (2016) [53] classified *Ghd7* alleles into three (strong function, weak function and non-function) and two (function and non-function) groups based on the sequence polymorphisms in the coding region, respectively. On the other hand, Lu et al. (2012) [38] analyzed 104 varieties (*O. sativa*) and three wild rice accessions (*O. rufipogon*) and found that 76 SNPs and six insertions and deletions within a 3932-bp DNA fragment of *Ghd7*. Among them, the functional C/T mutation in the promoter region was related to plant height probably by altering gene expression. In this study, we identified two novel alleles with functional differentiations in the promoter region of the *E1/Ghd7* allele. The interaction of these alleles with other flowering time genes except *Hd1* and *Hd5* have not yet been elucidated, additional detailed analysis of their effects on flowering time will contribute to fine-tuning of the flowering time well adapting to the climatic conditions in each area.

Photoperiod sensitivity is an important trait responsible for regional and seasonal adaptability of rice varieties. In this study, we detected two novel alleles at the *E1/Ghd7* locus. It is known that many other loci are involved in the photoperiod sensitivity pathway of flowering in rice. Recent studies showed that photoperiod sensitivity loci, *Se1/Hd1*, *OsPRR37* and *Ghd8*, each have a large allelic variation [49,54,55,56,57]. Therefore, analyzing the functional and inter-locus interactions should be advanced, which will lead to practice the fine-tuning of flowering time in rice breeding programs. In addition, we elucidated that the *E1* and *Ghd7* are the same locus. Until now, numerous genetic information has been accumulated for each locus (*E1*, *Ghd7*). For further genetic analyses of the *E1/Ghd7* locus, however, all information about *E1* and *Ghd7* should be available.

## 4. Materials and Methods 

### 4.1. Photoperiod Sensitivities of T65, T65m, and T65

The Taiwanese *japonica* rice variety “Taichung 65 (T65)”, and its isogenic line T65m and its near-isogenic line T65w were used. T65 harbors a photoperiod sensitivity allele *E1* at the *E1* locus, while T65m harbors a photoperiod-insensitivity allele *e1*, which is derived from the cross with Bozu5 [16,19,34,35]. T65w is a chromosome substitution line which was developed by introducing the *E1* region of *O. rufipogon* (W107) into the genetic background of T65 [36]. Five seeds were sown on field soil in a 3.6 L pot and covered with granulated soils. Seedlings were thinned to one plant per pot 14 days after sowing, and were grown under two photoperiod conditions, SD (12-h light/12-h dark) and LD (14-h light/10-h dark). Photoperiod treatments were conducted using two growth cabinets without temperature control. In addition to natural daylight (8:00–18:00), supplementary artificial light was used for the 12-h and 14-h light conditions. The degree of photoperiod sensitivity of each line was expressed as a difference of DH between SD and LD. The experiment was conducted by using five plants per line with three replications from 1 May to October 16. Heading date was recorded for each plant when the first panicle emerged from the sheath of the flag leaf.

### 4.2. Chromosomal Location of the E1 Locus

Two F_2_ populations from crosses of T65 × T65w, comprising 205 plants, and T65w × T65m, comprising 125 plants, were subjected to genetic analysis of heading date. They were grown in a paddy field of Kyoto University, Kyoto, Japan (35°01′ N). Seeds were sown on April 25 in 2007, and seedlings were transplanted on May 16 in 2007. The progeny test was conducted with 40 F_3_ lines (sowing on April 30 in 2008 and transplanting on May 21 in 2008). Each F_3_ line with approximately 25 plants was the progeny of an F_2_ plant randomly selected from the F_2_ population. To narrow down the candidate region of the *E1* locus, the F_4_ progenies derived from several F_3_ recombinants between RM1253 and RM3635 were cultivated (sowing on May 1 in 2009 and transplanting on May 22 in 2009).

### 4.3. Expression Analysis of E1/Ghd7

Plants of T65, T65m, and T65w were grown in a growth cabinet with a temperature controller under a LD condition (14.5-h light, 30 °C/9.5-h dark, 25 °C) at 70% relative humidity. Seedlings were grown on sand in 3.6 L pots (two plants/pot) with additional liquid fertilizer (Kimura’s B Culture Solution, Nippon Medical and Chemical Instruments Co., Ltd., Osaka, Japan). Thirty days before flowering, leaves were collected at 4-h intervals during that day. Total RNAs were extracted with Trizol reagent (Life Technologies Inc., Gaithersburg, Maryland, USA) according to the manufacturer’s protocols. Total RNA was subjected to DNA digestion by treatment with RNase-free DNase I (Takara Bio Inc.). The transcriptor first-strand cDNA synthesis kit (Roche Applied Science, Indianapolis, Indiana, USA) was used to reverse-transcribe cDNA from 1 µg of RNA using anchored-oligo (dT)18 primers. Real-time PCR analysis was performed by TaqMan PCR using a LightCycler 1.5 (Roche Applied Science) according to the manufacturer’s instructions. The primer sets of *Ghd7* and *UBQ* genes and Universal Probe Library probes of each gene were designed with ProbeFinder version 2.45 (Roche; https://www.roche-applied-science.com/). Primer and probe sequences are shown in Table S5. Expression analysis using the standard curve method was performed to determine the expression level of each gene. The relative expression level of each gene was calculated using *UBQ* gene. The RNA gene standards for the seven genes were applied to their plasmids prepared by the pGEM-T Easy Vector System (Promega Corp., Madison, Wisconsin, USA) using PCR amplicons from the total RNA of T65. 

### 4.4. Identification of the Genotype at the E1/Ghd7 Locus

To identify the *E1*/*Ghd7* genotype, we developed two DNA markers based on an SNP and a TE insertion. CAPS marker analysis was based on a nucleotide substitution from GAG (Glu) to TAG (stop codon) in exon 1, producing an additional restriction enzyme (*Spe* I) site. A pair of PCR primers (Ghd7_CAPS_F: 5′-CCAACTTGCCCTGTCTTCTT-3′, Ghd7_CAPS_R: 5′-AGCTGCTGCAAGCCAGTAAT-3′) was designed to amplify the 950 bp. PCR was performed with a 20 μL reaction mixture containing 2 μL template DNA, 10× PCR buffer, 25 mM MgCl_2_, 2 mM of each deoxyriboside-triphosphate (dNTP), 0.2 μL *Taq* DNA polymerase (5 U/μL), 4 μL of a 2.5 mM solution of each primer, and 3.2 μL H_2_O. PCR conditions were as follows: 94 °C for 5 min, followed by 35 cycles (1 min at 94 °C, 1 min at 60 °C, and 2 min at 72 °C) with a final extension of 7 min at 72 °C. The amplified products were digested with *Spe* Ι at 37 °C for 6 h. After digestion, the nonfunctional allele produced three fragments (81, 287, and 582 bp), whereas the functional allele produced two fragments (81 and 869 bp). Amplicons and digested amplicons were separated on 1% agarose gel. After electrophoresis, the gel was stained with ethidium bromide, and the DNA fragments were visualized under UV light. Insertion and deletion (INDEL) marker analysis was based on an 1897-bp copia-like TE insertion. A pair of PCR primers (Ghd7_INDEL_F: 5′-CGTTTCAGCAATAGCATTATGG-3′, Ghd7_INDEL_R: 5′-GCGGGTAGTCATCGAACAG-3′) was designed to amplify the 824 bp in the wild type and the 2721 bp in the insertion type. PCR was performed with a 20 μL reaction mixture containing 2 μL template DNA, 10× PCR buffer, 25 mM MgCl_2_, 2 mM of each dNTP, 0.2 μL *Taq* DNA polymerase (5 U/μL), 4 μL of a 2.5 mM solution of each primer, and 3.2 μL H_2_O. PCR conditions were as follows: 94 °C for 5 min, followed by 35 cycles (1 min at 94 °C, 1 min at 60 °C, and 2 min at 72 °C) with a final extension of 7 min at 72 °C. Amplicons were separated on 1% agarose gel. After electrophoresis, the gel was stained with ethidium bromide, and the DNA fragments were visualized under UV light.

### 4.5. Haplotype Patterns of the Chromosomal Region around the E1/Ghd7 Locus

Three DNA polymorphisms (two single-nucleotide substitutions and a 20 bp deletion) within the 100-kb chromosomal region surrounding *Ghd7* existed among 44 Hokkaido, 50 Japanese-core-collection [58] and 71 Aikoku-related varieties, which are considered to the derivatives of “Aikoku” variety based on their names. The Japanese rice core collection is a limited set of accessions representing, with a minimum set repetitiveness, the genetic diversity among Japanese rice varieties [58]. The two substitutions (Hap_SNP1 and Hap_SNP2) were detected by CAPS marker analyses. Hap_SNP1 harbors a G-to-T substitution, resulting in changing the recognition site of the restriction enzyme *Hpy188* I. Hap_SNP2 harbors a C-to-T substitution, resulting in changing the recognition site of *Hpy188* I. Primer pairs for Hap_SNP1 and Hap_SNP2 were designed to amplify 623 and 295 bp, respectively (Table S6). In addition, two polymorphism surveys of the *copia*-like TE insertion and the SNP in the first exon of *Ghd7* were performed. The CAPS and INDEL marker analyses to detect the insertion and SNP were described above.

## Supplementary Materials

The following are available online at https://www.mdpi.com/2223-7747/8/12/550/s1, Figure S1: Pedigree of the varieties, which harbor *e1-ret/ghd7-0ret* allele. Bold with underline indicates that the varieties harbor *e1-ret/ghd7-0ret* allele. Gray indicates varieties whose allele is unknown. Other indicates that the varieties harbor *e1/ghd7-0a* allele., Table S1: Frequency distributions of days to heading in F_3_ lines (T65 × T65w), Table S2: Frequency distributions of days to heading in F_3_ lines (T65m × T65w), Table S3: Lists of Hokkaido and Japanese core collection varieties used in this study, Table S4: Lists of Aikoku-related varieties used in this study, Table S5: Primer and probe sequences for expression analysis, Table S6 Primer sequences for haplotype analysis.
