# Supplementary material for: Allelic Differentiation at the E1/Ghd7 Locus Has Allowed Expansion of Rice Cultivation Area
Source: Plants (Basel). 2019 Nov 28;8(12):550. doi: 10.3390/plants8120550 (PMC6963527; doi:10.3390/plants8120550)
Supplement: Supplementary file 1 [file plants-08-00550-s001.pdf]

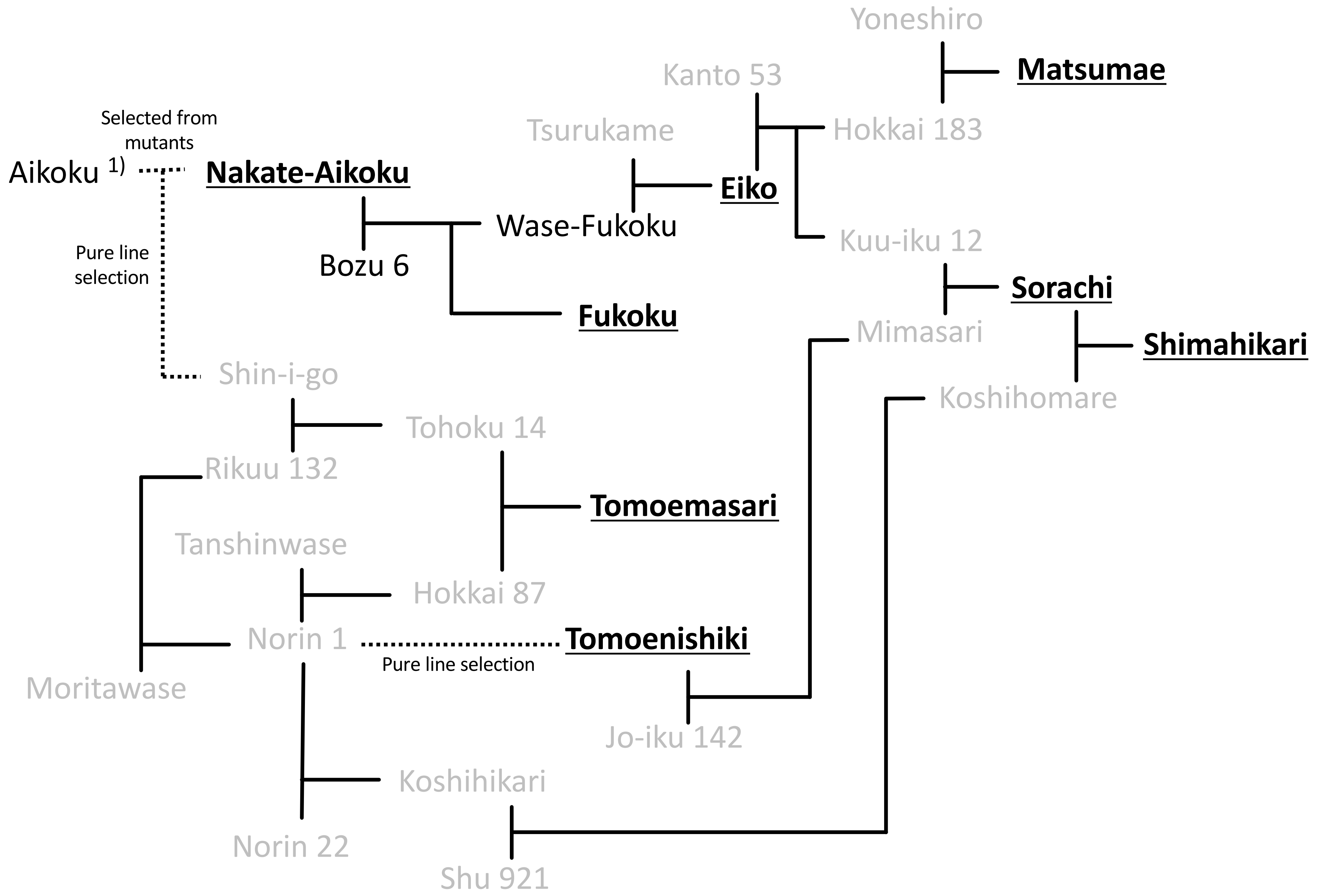

Figure S1. Pedigree of the varieties, which harbor *e1-ret/ghd7-0ret* allele. Bold with underline indicates that the varieties harbor *e1-ret/ghd7-0ret* allele. Gray indicates varieties whose allele is unknown. Other indicates that the varieties harbor *e1/ghd7-0a* allele.
[1] "Aikoku" includes multiple varieties with the mixture of the insertions.

Table S1. Frequency distributions of days to heading in F_3 lines (T65 × T65w)

Parents and F_3 lines	Genotype of parents and F_2 plants [a]	Days to heading																															Total	No. of F_3 lines observed [b]
		95	96	97	98	99	100	101	102	103	104	105	106	107	108	109	110	111	112	113	114	115	116	117	118	119	120	121	122	123	124	125		
T65	*E1E1*		7	5	7	1	1																										21	
T65w	*E1-rE1-r*																			2	3	2	2	3	4	5	1	2					24	
1						2	1	2	2	7	2	1				1																	18	
5							1		3	3	8	7	1	1	1																		25	
6					2		1	1	2	3	2	3	3																				17	
7				1		1	5	2	3	6		1	2	1	2																		24	
12							3	1	3	7		3	3	2		1																	23	
14										1	1	4	1		1																		8	
16	*E1E1*								1	3	5	5	4	2	1	1																	22	13
17						1	3	1	6	4	3	2	1	1																			22	
18								2		2	3	3	2	2		1																	13	
22									3	3	4	5	5	1		1																	22	
23						1		2	2	3	2				1																		11	
25								1	1	4	5	3	2	4	2																		22	
26							1	1	3	7	3	3	1			1																	20	
2								1		1		1	1	1	1		2	1	4	1	2		3		1	1							21	
4												3					2	1	6	2		1	3	2									20	
8										1		3	2	1	3		2	1	2	2		2	2		1								22	
9													2		5				1	2			2	1		1	2	1					17	
10								1	1		2	3					2	2	5	1	3	1		1	1	1							24	
11										1		1	2	3	1		1	2	2	5	2	1	2		1	1							25	
13	*E1E1-r*								2		1		1	3	1		2	2	1	1	3		1	4	1								23	14
19										1	1	1	1				4	2	2			1	1										14	
24											2	2	1	2	1		1	3	2	5	1	1				2							23	
29									3		1		1				2	5	2	1	2	1		1									19	
30														2		1	4	2	4	2	4			1	1								21	
33											1	2		1	1		2	1	3	2				5	2	1	1	1					23	
37					1		1	1	3	1	1				3	1	2	1	2			1	2	2									22	
39										1	1		2	3	1	1	2	3		1	4	1	2	2									24	
3																						4	7	8	2	2	1						24	
20																				1	1		2	3	5	4	3	1					20	
21																			2			2	3	5	4	1	3		1				20	
27																		1	3	1	3	1	5	2	2	1		1					20	
28																				3		4	3	7	6	1							24	
31	*E1-rE1-r*																				1	2		1	4	7	5	2	1				23	11
32																			1	1		3	2	6	7	3							23	
34																							1		4	4	4	3	5	1	2		24	
35																	1		2		5	3	2	4	1								18	
36																			1	1	1	1	5	8	2	3							22	
40																						1	1	1	3	6	6	5	1				24	

[a] *E1* and *E1-r* are early and late heading alleles of *E1* locus, respectively.

[b] *E1E1* : *E1E1-r* : *E1-rE1-r* = 13:14:11, χ^2 = 8.904, P = 0.012)

Table S2. Frequency distributions of days to heading in F_3 lines (T65m × T65w)

Parents and F_3 lines	Genotype of parents and F_2 plants [a]	Days to heading																															Total	No. of F_3 lines observed [b]
		95	96	97	98	99	100	101	102	103	104	105	106	107	108	109	110	111	112	113	114	115	116	117	118	119	120	121	122	123	124	125		
T65m	*e1e1*				1		8	5	5																								19	
T65w	*E1-rE1-r*																			2	3	2	2	3	4	5	1	2					24	
8			1		2	2	1	6	7	4																							23	
18			1			4	6	2	7	2		2																					24	
20					1	1	3	6	4	1	2	1	1	1																			20	
21			3	2	3	4	2	2	4	1																							21	
28	*e1e1*				2	4	4	5	4	1																							20	10
29					3	5		7	6	1		1																					23	
34					4	2	1	4	8	2		1																					22	
35		1	1		5	1	1																										9	
40									2						1																		3	
52			1		1		4	5	6	1	2																						20	
3								1				1			3	2	2	3	3			3	1	1	2			1					23	
4									2	4	1	1	1				1		3	4	1	1		2		1		3					25	
7								2				1		1	1				3	1	4	2	2	2		2							21	
9				1		1		1	4	1					2		3	3	1	3	3	1	1										25	
10							1	2	2							2	2	3	2	1		5	1	1									22	
11									1		1				1		1	4	1	1	2	1	1		1								15	
12							1		2	1	4						1	1	1	2	4				2		1						20	
15					1			3	1						1		2		1		1	1						1					12	
16									2		2		1			1	2	1	2	2	2		1	1	1	4							22	
19							1	1							1		2	2			1		1	1	1								11	
23	*e1E1-r*							2	1	1		1			2	1	2	2	3	2		3	1	1									22	21
24						1	1	1	1	1				2	1	2	3		5		1			2		1							22	
27					2		2								3	1	4	3	1			1	1	1		1							20	
33								1							1	1		1	2				2		1								9	
36					1	1		1	2	2				1	1	3	1	3	1	2	1		2	2									24	
37							1	3				1			2	3	1	3	1	2	2	1	1	1		1							23	
38								2	1	2	2						1	4	6	1	1	1		1		1							23	
42					1					2	1		1	1			2	2	5	2	2			3		2	1						25	
51					1		1	1	3							1	3	2	4		1	2	1	1	1	1							23	
54					1	1			4	1			1			1	1	2	2	1	1			1									17	
55						1	1	1	2			1				2	6	1	3	2			1										21	
2																			1	1	3	3	5	6	1	2	3						25	
6																					7	7	3	4	1		1						23	
31																	1					1		7	7	2	1		2				21	
32																					4	2	1	5	3	3	4						22	
39	*E1-rE1-r*																			5	5	2	4	3	2	2							23	9
41																	1		2	2	2	3	5	2	2	2							21	
53																		1	4	2	5	6	5										23	
56																			1		1	3	5	7	3	4							24	
57																							2	5	6	6							19	

[a] *e1* and *E1-r* are early and late heading alleles of *E1* locus, respectively.

[b] *e1e1* : *e1E1-r* : *E1-rE1-r* = 9:21:10, χ^2 = 0.150, P = 0.928)

Table S3. Lists of varieties used in this study

Group	Name	Origin	ID No. [1)]	landrace/improved	Subspecies	Haplotype around *E1* locus [2)]	Allele of *E1/Ghd7* locus [2)]	Days to heading [2), 3)]
Hokkaido varieties	Akabozu	Hokkaido	-	improved	Japonica	Hap1	*e1/ghd7-0a*	69.9
	Akage	Hokkaido	-	landrace	Japonica	Hap1	*e1/ghd7-0a*	68.4
	Akatsuki	Hokkaido	-	improved	Japonica	Hap1	*e1/ghd7-0a*	65.3
	Bozu	Hokkaido	-	landrace	Japonica	Hap1	*e1/ghd7-0a*	68.4
	Bozu 6	Hokkaido	-	improved	Japonica	Hap1	*e1/ghd7-0a*	67.2
	Dohoku 5	Hokkaido	-	improved	Japonica	Hap1	*e1/ghd7-0a*	65.1
	Eiko	Hokkaido	-	improved	Japonica	Hap3	*e1-ret/ghd7-0ret*	65.6
	Fukoku	Hokkaido	-	improved	Japonica	Hap3	*e1-ret/ghd7-0ret*	66.0
	Fukuyuki	Hokkaido	-	improved	Japonica	Hap1	*e1/ghd7-0a*	68.1
	Hashiribozu	Hokkaido	-	improved	Japonica	Hap1	*e1/ghd7-0a*	65.1
	Hayakaze	Hokkaido	-	improved	Japonica	Hap1	*e1/ghd7-0a*	64.5
	Hayakogane	Hokkaido	-	improved	Japonica	Hap1	*e1/ghd7-0a*	67.1
	Hayamasari	Hokkaido	-	improved	Japonica	Hap1	*e1/ghd7-0a*	65.4
	Hokkai 126	Hokkaido	-	improved	Japonica	Hap1	*e1/ghd7-0a*	63.8
	Hokkai 242	Hokkaido	-	improved	Japonica	Hap1	*e1/ghd7-0a*	63.5
	Hokkai 244	Hokkaido	-	improved	Japonica	Hap1	*e1/ghd7-0a*	65.3
	Hokuto	Hokkaido	-	improved	Japonica	Hap1	*e1/ghd7-0a*	64.2
	Iburiwase	Hokkaido	-	landrace	Japonica	Hap1	*e1/ghd7-0a*	65.3
	Ishikari	Hokkaido	-	improved	Japonica	Hap1	*e1/ghd7-0a*	66.0
	Ishikarisirage	Hokkaido	-	improved	Japonica	Hap1	*e1/ghd7-0a*	67.8
	Jo-iku 100	Hokkaido	-	improved	Japonica	Hap1	*e1/ghd7-0a*	67.8
	Jo-iku 272	Hokkaido	-	improved	Japonica	Hap1	*e1/ghd7-0a*	64.4
	Kirara 397	Hokkaido	-	improved	Japonica	Hap1	*e1/ghd7-0a*	71.3
	Kitaake	Hokkaido	-	improved	Japonica	Hap1	*e1/ghd7-0a*	66.4
	Kitahikari	Hokkaido	-	improved	Japonica	Hap1	*e1/ghd7-0a*	68.3
	Kitakogane	Hokkaido	-	improved	Japonica	Hap1	*e1/ghd7-0a*	66.6
	Kiyokaze	Hokkaido	-	improved	Japonica	Hap1	*e1/ghd7-0a*	65.1
	Kyowa	Hokkaido	-	improved	Japonica	Hap1	*e1/ghd7-0a*	67.7
	Matsumae	Hokkaido	-	improved	Japonica	Hap3	*e1-ret/ghd7-0ret*	68.3
	Megurosakaemoti	Hokkaido	-	improved	Japonica	Hap1	*e1/ghd7-0a*	71.5
	Norin 11	Hokkaido	-	improved	Japonica	Hap1	*e1/ghd7-0a*	63.0
	Norin 15	Hokkaido	-	improved	Japonica	Hap1	*e1/ghd7-0a*	63.8
	Norin 33	Hokkaido	-	improved	Japonica	Hap1	*e1/ghd7-0a*	64.6
	Sakigake	Hokkaido	-	landrace	Japonica	Hap1	*e1/ghd7-0a*	65.7
	Sasahonami	Hokkaido	-	improved	Japonica	Hap1	*e1/ghd7-0a*	66.6
	Shimahikari	Hokkaido	-	improved	Japonica	Hap3	*e1-ret/ghd7-0ret*	69.3
	Shinei	Hokkaido	-	improved	Japonica	Hap1	*e1/ghd7-0a*	73.4
	Shiokari	Hokkaido	-	improved	Japonica	Hap1	*e1/ghd7-0a*	65.7
	Sorachi	Hokkaido	-	improved	Japonica	Hap3	*e1-ret/ghd7-0ret*	73.2
	Tokachikuroge	Hokkaido	-	improved	Japonica	Hap1	*e1/ghd7-0a*	60.8
	Tomomasari	Hokkaido	-	improved	Japonica	Hap3	*e1-ret/ghd7-0ret*	73.1
	Tomonishiki	Hokkaido	-	improved	Japonica	Hap3	*e1-ret/ghd7-0ret*	72.4
	Wasebozu	Hokkaido	-	landrace	Japonica	Hap1	*e1/ghd7-0a*	62.8
	Wasehukoku	Hokkaido	-	improved	Japonica	Hap1	*e1/ghd7-0a*	66.4

Table S3. Lists of varieties used in this study

Group	Name	Origin	ID No. 1)	landrace/improved	Subspecies	Haplotype around *E1* locus 2)	Allele of *E1/Ghd7* locus 2)	Days to heading 2), 3)
Japanese core collection	Gaisen Mochi	Japan (Unknown)	JRC 01	landrace	Tropical Japonica	N. A.	*E1/Ghd7-2*	103.5
	Hinode	Kinki	JRC 03	improved	Tropical Japonica	Hap4	*E1/Ghd7-2*	86.3
	Senshou	Tokyo	JRC 04	landrace	Tropical Japonica	Hap4	*E1/Ghd7-2*	87.0
	Yamada Bake	Kagoshima	JRC 05	landrace	Tropical Japonica	Hap4	*E1/Ghd7-2*	94.5
	Kaneko	Kantotozan	JRC 06	landrace	Tropical Japonica	Hap4	*E1/Ghd7-2*	84.1
	Iruma Nishiki	Saitama	JRC 07	landrace	Tropical Japonica	Hap4	*E1/Ghd7-2*	84.9
	Okka Modoshi	Japan (Unknown)	JRC 08	landrace	Tropical Japonica	Hap4	*E1/Ghd7-2*	101.0
	Hirayama	Tokyo	JRC 10	landrace	Tropical Japonica	Hap4	*E1/Ghd7-2*	84.3
	Kahei	Kagoshima	JRC 11	landrace	Tropical Japonica	Hap4	*E1/Ghd7-2*	N. A.
	Oiran	Kumamoto	JRC 12	landrace	Tropical Japonica	Hap4	*E1/Ghd7-2*	91.8
	Bouzu Mochi	Oita	JRC 13	landrace	Tropical Japonica	Hap4	*E1/Ghd7-2*	92.8
	Meguro Mochi	Kantotozan	JRC 14	landrace	Tropical Japonica	Hap4	*E1/Ghd7-2*	103.0
	Akage	Hokkaido	JRC 17	landrace	Japonica	Hap1	*e1/ghd7-0a*	N. A.
	Hassokuho	Japan (Unknown)	JRC 18	landrace	Japonica	Hap4	*E1/Ghd7-2*	93.0
	Wataribune	Shiga	JRC 19	landrace	Japonica	Hap4	*E1/Ghd7-2*	109.4
	Hosogara	Aomori	JRC 20	landrace	Japonica	Hap4	*E1/Ghd7-2*	78.5
	Akamai	Kochi	JRC 21	landrace	Japonica	Hap4	*E1/Ghd7-2*	96.4
	Mansaku	Nagano	JRC 22	landrace	Japonica	Hap4	*E1/Ghd7-2*	89.3
	Ishijiro	Toyama	JRC 23	landrace	Japonica	Hap2	*E1/Ghd7-2*	98.8
	Joushuu	Yamagata	JRC 24	landrace	Japonica	Hap4	*E1/Ghd7-2*	92.7
	Dango	Japan (Unknown)	JRC 25	landrace	Japonica	Hap4	*E1/Ghd7-2*	109.6
	Aikoku	Fukui	JRC 26	landrace	Japonica	Hap3	*e1-ret/ghd7-0ret*	91.3
	Ginbouzu	Ishikawa	JRC 27	landrace	Japonica	Hap4	*E1/Ghd7-2*	102.3
	Shinriki Mochi	Kumamoto	JRC 28	landrace	Japonica	Hap4	*E1/Ghd7-2*	111.3
	Shichimenchou Mochi	Japan (Unknown)	JRC 29	landrace	Japonica	Hap4	*E1/Ghd7-2*	107.4
	Morita Wase	Yamagata	JRC 30	landrace	Japonica	Hap4	*E1/Ghd7-2*	82.0
	Kameji	Shimane	JRC 31	landrace	Japonica	Hap4	*E1/Ghd7-2*	106.5
	Omachi	Okayama	JRC 32	landrace	Japonica	Hap4	*E1/Ghd7-2*	114.0
	Shinriki	Hyogo	JRC 33	landrace	Japonica	Hap4	*E1/Ghd7-2*	110.0
	Kyoutoasahi	Kyoto	JRC 34	landrace	Japonica	Hap4	*E1/Ghd7-2*	110.4
	Kabashiko	Miyazaki	JRC 35	landrace	Japonica	Hap4	*E1/Ghd7-2*	106.9
	Sekiyama	Aomori	JRC 36	landrace	Japonica	Hap4	*E1/Ghd7-2*	78.8
	Shinyamadaho 2	Hyogo	JRC 37	landrace	Japonica	Hap2	*E1/Ghd7-2*	108.8
	Nagoya Shiro	Akita	JRC 38	landrace	Japonica	Hap5	*E1/Ghd7-2*	91.7
	Shiroine (Kemomi)	Tokushima	JRC 39	landrace	Japonica	Hap4	*E1/Ghd7-2*	109.8
	Akamai	Nagasaki	JRC 40	landrace	Indica	Hap2	*E1/Ghd7-2*	116.4
	Akamai	Tokushima	JRC 41	landrace	Indica	Hap2	*E1/Ghd7-2*	110.9
	Touboshi	Kagoshima	JRC 42	landrace	Indica	N. A.	N. A.	N. A.
	Akamai	Kantotozan	JRC 43	landrace	Indica	Hap4	*E1/Ghd7-2*	98.6
	Karahoushi	Kagoshima	JRC 44	landrace	Indica	N. A.	N. A.	N. A.
	Hiyadachitou	Yamagata	JRC 45	landrace	Japonica	Hap4	*E1/Ghd7-2*	78.6
	Fukoku	Hokkaido	JRC 46	landrace	Japonica	Hap3	*e1-ret/ghd7-0ret*	N. A.
	Okabo	Japan (Unknown)	JRC 47	landrace	Japonica	Hap4	*E1/Ghd7-2*	106.4
	Hakamuri (Yokoyama)	Kagoshima	JRC 48	landrace	Japonica	Hap4	*E1/Ghd7-2*	96.0
	Rikutou Rikuu 2	Japan (Unknown)	JRC 49	landrace	Japonica	N. A.	*E1/Ghd7-2*	87.7
	Himenomochi	Akita	JRC 50	improved	Japonica	Hap2	*E1/Ghd7-2*	82.8
	Shinshuu	Nagano	JRC 51	landrace	Japonica	Hap2	*E1/Ghd7-2*	85.1
	Aichiasahi	Aichi	JRC 52	improved	Japonica	Hap4	*E1/Ghd7-2*	109.8
	Raiden	Kantotozan	JRC 53	landrace	Japonica	Hap4	*E1/Ghd7-2*	113.7
	Houmanshinden Ine	Kagoshima	JRC 54	landrace	Japonica	Hap4	*E1/Ghd7-2*	120.0

1): ID number of the Japanese core collection

2): N. A. : Not Available data

3): Days to heading is from sowing (April 30, 2010) in Kyoto.

Table S4. Lists of varieties used in this study

Name	Origin	NIAS gene bank ID	landrace/improved	Haplotype around *E1* locus	Allele of *E1/Ghd7* locus
Riku Aikoku	Kantotozan	4510	landrace	Hap4	*E1/Ghd7-2*
Riku Aikoku	Kantotozan	4713	landrace	Hap4	*E1/Ghd7-2*
Hakubou Aikoku	Gunma	4774	landrace	Hap4	*E1/Ghd7-2*
Aikoku	Gunma	4982	landrace	Hap4	*E1/Ghd7-2*
A Aikoku 2	Tohoku	5247	improved	Hap4	*E1/Ghd7-2*
Aikoku	Akita	5419	landrace	Hap5	*E1/Ghd7-2*
Gokuwaseaikoku	Akita	5777	improved	Hap4	*E1/Ghd7-2*
Aikokudaikoku	Akita	5792	improved	Hap3	*e1-ret/ghd7-0ret*
Aikokudaikokumochi A	Akita	5793	improved	Hap3	*e1-ret/ghd7-0ret*
Aikokudaikokumochi B	Akita	5794	improved	Hap4	*E1/Ghd7-2*
Aikokutarehatou	Akita	5795	improved	Hap3	*e1-ret/ghd7-0ret*
Aikokuwaitou	Akita	5796	improved	Hap3	*e1-ret/ghd7-0ret*
Aikokumitsuryuutou	Akita	5810	improved	Hap3	*e1-ret/ghd7-0ret*
Nakatemubouaikoku	Nagano	5998	improved	Hap3	*e1-ret/ghd7-0ret*
Nakateaikoku 3	Ibaraki	5999	improved	Hap3	*e1-ret/ghd7-0ret*
Waitouaikoku	Tohoku	6044	landrace	Hap3	*e1-ret/ghd7-0ret*
Aikokutarehatou	Tohoku	6047	landrace	Hap3	*e1-ret/ghd7-0ret*
Bouzuaikoku	Yamagata	6133	landrace	Hap3	*e1-ret/ghd7-0ret*
Rikuuaikoku 20	Akita	6158	improved	Hap3	*e1-ret/ghd7-0ret*
Aikoku	Fukui	6365	landrace	Hap3	*e1-ret/ghd7-0ret*
Aikoku	Fukui	6369	landrace	Hap3	*e1-ret/ghd7-0ret*
Aikoku 70	Niigata	6442	improved	Hap3	*e1-ret/ghd7-0ret*
Aikoku	Miyagi	6730	landrace	Hap3	*e1-ret/ghd7-0ret*
Nakateaikoku	Yamagata	6744	improved	Hap3	*e1-ret/ghd7-0ret*
Nakateshinaikoku	Akita	6752	improved	Hap3	*e1-ret/ghd7-0ret*
Waseaikoku	Yamagata	6753	landrace	Hap5	*E1/Ghd7-2*
Aikoku 1	Miyagi	6757	improved	Hap4	*E1/Ghd7-2*
Aikoku	Miyagi	6768	landrace	Hap3	*e1-ret/ghd7-0ret*
Aikokuibaraki 1	Ibaraki	6771	improved	Hap3	*e1-ret/ghd7-0ret*
Aikokuibaraki 2	Ibaraki	6822	improved	Hap3	*e1-ret/ghd7-0ret*
Akitawaseaikoku 5	Akita	6915	improved	Hap4	*E1/Ghd7-2*
Waseaikoku	Miyagi	6927	improved	Hap3	*e1-ret/ghd7-0ret*
Waseaikoku 2	Miyagi	6928	improved	Hap3	*e1-ret/ghd7-0ret*
Aikokuho	Miyagi	7013	landrace	Hap3	*e1-ret/ghd7-0ret*
Kairyouaikoku 17	Osaka	7053	improved	Hap4	*E1/Ghd7-2*
Waseaikoku 96	Chiba	7054	improved	Hap3	*e1-ret/ghd7-0ret*
Nakateaikoku 90	Chiba	7055	improved	Hap3	*e1-ret/ghd7-0ret*
Toukyoumubouaikoku	Tokyo	7058	landrace	Hap3	*e1-ret/ghd7-0ret*
Aikoku 3	Tochigi	7084	improved	Hap3	*e1-ret/ghd7-0ret*
Aikokusai 1	Saitama	7085	improved	Hap3	*e1-ret/ghd7-0ret*
Aikoku 20	Akita	7095	improved	Hap4	*E1/Ghd7-2*
Aikoku 6	Gunma	7096	improved	Hap3	*e1-ret/ghd7-0ret*
Waseaikoku 30	Gunma	7097	improved	Hap3	*e1-ret/ghd7-0ret*
Shizuokaaikoku 1	Shizuoka	7189	improved	Hap3	*e1-ret/ghd7-0ret*
Aikoku 5	Fukushima	7198	improved	Hap3	*e1-ret/ghd7-0ret*
A Aikoku 1	Miyagi	7228	improved	Hap2	*E1/Ghd7-2*
Aikoku 3	Tochigi	7332	improved	Hap3	*e1-ret/ghd7-0ret*
Aikoku A	Kanagawa	7556	landrace	Hap4	*E1/Ghd7-2*
Choubouaikoku	Kantotozan	7560	landrace	Hap2	*E1/Ghd7-2*
Shinkane/Aikoku	Kanagawa	7576	improved	Hap2	*E1/Ghd7-2*
Kairyouaikokudaikokugata 1	Miyagi	7621	improved	Hap3	*e1-ret/ghd7-0ret*

Kairyouaikokudaikokugata 2	Miyagi	7622	improved	Hap3	*e1-ret/ghd7-0ret*
Banaikoku	Gifu	9192	landrace	Hap4	*E1/Ghd7-2*
Aikoku 68	Mie	9277	improved	Hap3	*e1-ret/ghd7-0ret*
Aikoku 1 (Kyouto)	Kyoto	9285	improved	Hap3	*e1-ret/ghd7-0ret*
Aikoku	Shiga	9310	landrace	Hap3	*e1-ret/ghd7-0ret*
Aikokumochi	Mie	9412	landrace	Hap4	*E1/Ghd7-2*
Aikoku B	Osaka	9513	improved	Hap3	*e1-ret/ghd7-0ret*
Aikoku C	Osaka	9514	improved	Hap3	*e1-ret/ghd7-0ret*
Aikoku D	Osaka	9515	improved	Hap3	*e1-ret/ghd7-0ret*
Aikoku F	Osaka	9516	improved	Hap2	*E1/Ghd7-2*
Aikoku G	Osaka	9517	improved	Hap3	*e1-ret/ghd7-0ret*
Aikoku	Tokushima	9650	landrace	Hap3	*e1-ret/ghd7-0ret*
Aikoku 1	Shimane	9823	improved	Hap3	*e1-ret/ghd7-0ret*
Aikoku 2	Shimane	9824	improved	Hap3	*e1-ret/ghd7-0ret*
Aikoku	Kantotozan	10993	landrace	Hap4	*E1/Ghd7-2*
Aikoku (4X)	Fukuoka	10994	improved	Hap4	*E1/Ghd7-2*
Mubouaikoku	Ibaraki	14931	landrace	Hap3	*e1-ret/ghd7-0ret*
Kairyouaikoku	Osaka	15007	improved	Hap2	*E1/Ghd7-2*
Waseaikoku 3	Kanagawa	15051	landrace	Hap3	*e1-ret/ghd7-0ret*
W (Waseaikoku)	Akita	37731	unknown	Hap3	*e1-ret/ghd7-0ret*

Table S5. Primer and probe sequences and probes ID for quantitative RT-PCR

Gene	Forward sewuence	Reverse sewuence	product size	catalog number of probe[1]	probe sequence
Ghd7	GAGGGAGGCCAAGCTGAT	ACCGGATTTGCTTCTCGTAG	68	#35 4687680001	agaagagga
Ubiquitin	CTCCGTGGCGGTATCATC	TCTTCTTGCGGCAGTTGAC	124	#124 4693582001	no data

[1] The catalog number of probe is based on the Universal Probe Library designed with ProbeFinder version 2.45 (Roche; https://www.roche-applied-science.com/).

Table S6. Primers used for haplotype analysis

Name of primers	Forward primer	Reverse primer	anneling temperture (℃)	Product size (bp)	SNP	Enzyme[1)]
Haplotype_INDEL	CCCCCAAACATTTTCAGAAC	CTGCACCTAATCCGAATTCA	55	234[2)]	-	-
Haplotype_SNP1	ATGTCATGCGGTGGGATTG	CCGAAGGCCAAACTAGGAAG	62	623	G/T[3)]	Hpy188I
Haplotype_SNP2	GCTTGAAGATTGGGAGCTTG	CACTAGAAGGAGTTCACCTTTTCTTC	55	295	C/T[3)]	Hpy188I

[1)] Enzyme is used in CAPS marker analysis.

[2)] The number indicates PCR product size without the 20bp deletion.

[3)] The nucleotides indicate the Nipponbare and EG5 genome sequence (left) and Kirara397 genome sequence (right).
